# Concurrent bilateral testicular tumors with different histopathology: A case report

**DOI:** 10.22088/cjim.11.4.446

**Published:** 2020

**Authors:** Ghasem Rostami, Hamid Shafi, Mohammad Ranaee, Samaneh Saeedfar, Fatemeh Mahmoudlou

**Affiliations:** 1 Student Research Committee, Babol University of Medical Sciences, Babol, Iran; 2 Cancer Research Center, Health Research Institute, Babol University of Medical Sciences, Babol Iran; 3 Clinical Research Development Center, Shahid Beheshti Hospital, Babol University of Medical Sciences, Babol, Iran; 4 Department of Urology, School of Medicine, Babol University of Medical Sciences, Babol, Iran; 5 Infertility and Health Reproductive Research Center, Health Research Institute, Babol University of Medical Sciences, Babol, Iran; 6 Department of Pathology, School of Medicine, Babol University of Medical Sciences, Babol, Iran

**Keywords:** Testis, Tumors, Histology

## Abstract

**Background:**

Bilateral synchronous testis germ cell tumors with different histopathology are not common.

**Case Presentation:**

Here, a case of 27-year-old male who is reported presented with bilateral testicular swellings. There was a high α-fetoprotein level with bilateral lesions on scrotal Ultrasonography. Bilateral orchiectomy was performed. According to pathology report, there was a right testicular seminoma and left testicular mixed germ cell tumor composed of seminoma, yolk sac tumor and embryonal carcinoma.

**Conclusion:**

He received one cycle of chemotherapy with BEP regimens.

Testis tumors are rare and account for 1-1.5 % of all malignancies occurring in men. Incidence rates increased between 1992-2009 (1, 2). Testicular tumor is the most prevalent type of cancer among men between the ages of 15-44 (3). Seminomas and non-seminomas are the most frequent testicular germ cell tumors with a peak incidence at 35 and 25 years, respectively (4). The probability of occurrence of BGCT is estimated between 0.5% and 7% which is rare, most of the cases present metachronous tumors and about 15-20% present synchronous tumors (5). There are no connections through lymphatic or vascular paths between the testes, so synchronous tumors develop independently as two primary tumors (6). In this paper, a case of BGCT with non-seminoma in the left side and right side seminoma is presented. 

## Case Presentation

A 27 year old single male presented to our patient department with complaint of one month testicular swelling with left side preference. He has no complaints of pain in the testicles and abdomen. No problem in erectile function. He had no problems during puberty. He had no history of smoking and use of opioid in social habits. There is no family history and no evidence of risk factor for testicular cancer such as cryptorchidism or congenital abnormalities in the patient. Physical examination specified the left side testis with twice the normal volume swelling and without tenderness. The size of right side testis has increased slightly. Laboratory workup revealed azoospermia and an elevated a-FP (258.4IU/mL), and b-hcG (3.12mIU/mL) within normal levels. On ultrasound study the testes have 50mm×30mm in right side and 72mm×50mm in left side dimensions. No hydrocele was seen. Images of the right testis demonstrated 38mm×27mm hypoecho mass that accounting for (occupying) three-quarters of the volume of the parenchyma. Images of the left testis demonstrated approximately 70mm×47mm mixed echogenic mass, comprises almost the entire volume of the testis. Epididymis have normal parenchymal dimensions and echoes.

**Fig. 1:**
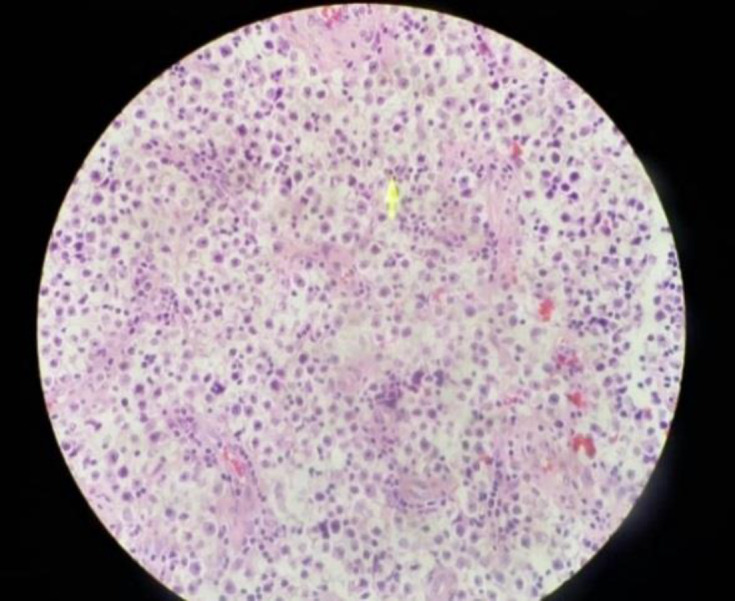
Seminoma of the right testis. Sheets of tumor cells with pale-staining cytoplasm and an associated lymphoid infiltrate

The ultrasound study of abdomen and pelvis showed no abnormality. The abdomen CT scan was not indicative of enlargement of the lymph nodes of retroperitoneum. The chest x-ray did not show evidence of metastasis. The patient underwent bilateral radical orchiectomy. Right testicular mass, excisional biopsy, for frozen section and intra-operative diagnosis, consists of a piece of creamy colored soft tissue specimen, with homogenous appearance, measured 45×35×22 mm in the largest diameters. Cryo and permanent sections of right testis mass, confirmed invasive, classic type seminoma that limited to the testis with intratubular and invasion to lympho-vascular tissues (tumor stage: at-least PT2) (fig. 1).

**Fig. 2:**
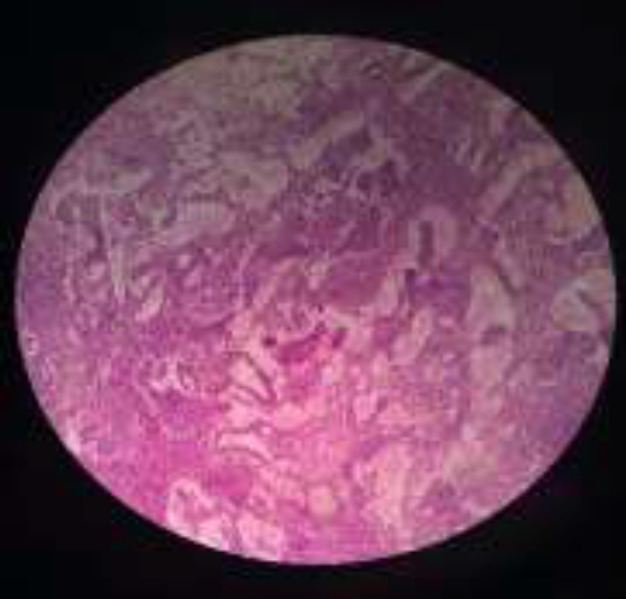
Yolk sac tumor of the left testis. Lace-like (reticular) pattern of tumor cells

**Fig. 3:**
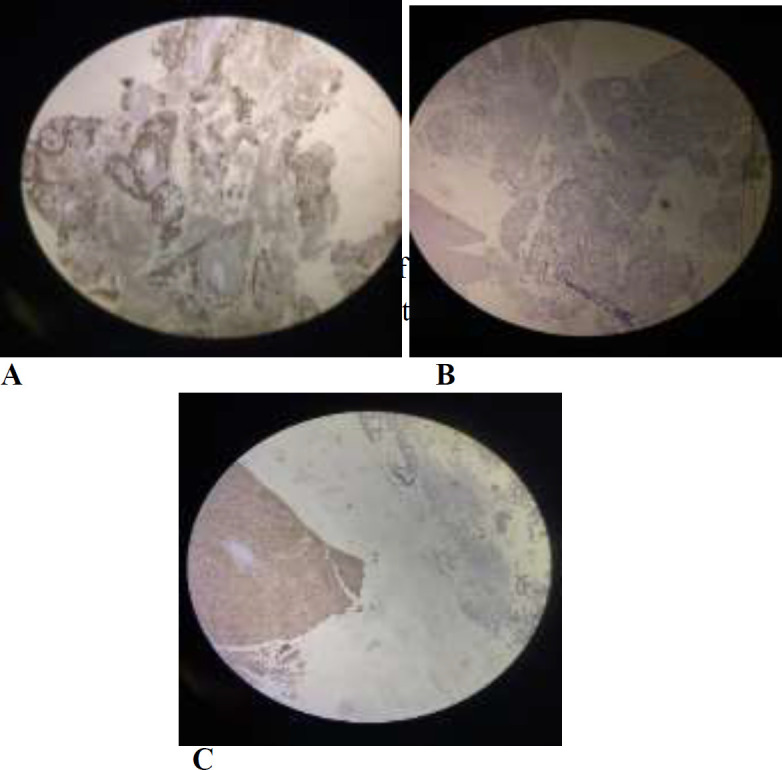
Immunohistochemical staining; (a) AE1/AE3 Staining pattern. (b) CD30 Staining pattern. (c) CD117 Staining pattern

In the left testis, in cut sections, almost all testicular volume replaced by tumoral tissue with heterogeneous appearance, containing solid and cystic, hemorrhagic and necrotic areas, measured 80×50×40 mm. the left side tumor was a malignant GCT with component of invasive, classic type seminoma (50-60%), yolk sac tumor (40-50%) and embryonal carcinoma (about 10%) (fig. 2). Tumor extended to rete testis, tunica albuginea and tunica vaginalis. Epididymis was not involved. Intratubular and lympho-vascular invasion was present (tumor stage: PT2). Immunocytochemical study with AE1/AE3, CD30 and CD117 markers demonstrated a positive reaction (fig. 3 a,b,c). The patient was discharged without problems and complications one day after surgery. The elevated a-FP on the postoperative measurement decreased to lower values (6,05mIU/mL). The patient was advised to go to an oncologist and was submitted to one cycle of adjuvant chemotherapy with bleomycin, etoposide, and cisplatin (BEP). As the follow-up physical examination, serum markers, chest X ray, and CT of the abdomen, were checked. Six months after the orchiectomy there was no residual tumor or recurrence, neither local nor systematic. Finally the patient is under a follow-up by an endocrinologist for the long-term management regarding the testosterone replacement therapy and he has been started on topical testrogel for life.

## Discussion

Bilateral testicular germ cell tumor is a rare event, accounting for approximately 1-3% of all testicular germ cell tumors (7). Tumors of germ cell type can occur in testis, retroperitoneum, mediastinum and pineal gland (8). The serum levels of tumor markers LDH, alpha-fetoprotein and beta-HCG are diagnostic (9). Common metastasis of primary testicular germ cell tumor is to retroperitoneal lymph nodes, particularly the paraaortic lymph nodes on the left and the interaortocaval lymph nodes on the right (10). Choriocarcinoma spreads hematogenously (11).

The incidence of bilateral testicular cancer is about 2-5%. The majority occurs metachronously. Synchronous bilateral testis tumor accounts for 0.5-1% of all testicular cancers and most of them are of identical histology (12, 13). The most prevalent histolopathologic type is seminoma, comprising around 80% of tumors and the remaining are mixed teratomas, yolk sac tumors and both pure and mixed embryonal cell carcinomas (14). A recent study of 118 cases with bilateral testicular cancer has demonstrated that only 11 patients presented with different histological characteristics (15). In 2003, Coli et al. reported the 43th case of synchronous BGCT with different histopathology and only six patients presented with synchronous seminomatous and mixed GCTs. according to their review of literature (6).

Seminoma, accounting for 35%-50% of all germ cell tumors, is the most prevalent germ cell tumor. The most common histologic type which is present in mixed germ cell tumors is embryonal carcinoma (87%), followed by teratoma (50%), yolk sac (44%) and choriocarcinoma (8%) (16). In this case, scrotumal ultrasonography revealed bilateral synchronous testicular mass. Post- operation pathology report revealed right side pure seminoma and left side mixed germ cell tumor. The risk factors predisposing testicular cancer are: age, cryptorchidism, family history of testicular cancer, Klinefelter's syndrome, personal history of testicular cancer, congenital abnormalities and infertility (17). The cellular morphology of seminoma is similar to that of primitive germ cells. The cells are relatively the same-shaped with well-defined borders, clear cytoplasm and nuclei with one or more prominent nucleoli (18).

Bilateral orchiectomy is still the treatment of choice for synchronous bilateral testicular tumors. The treatment depends on the tumor stage and the nonseminomatous components of the testes (6, 16, 19). In early stages of tumors, chemotherapy and testis-sparing surgery can be considered due to their endocrinological and psychological advantages (6). A study of 73 cases of bilateral testicular germ cell tumors who underwent tumor enucleation and radiation therapy demonstrated that 72 (98.6%) survived without evidence of disease after a 7 year follow-up. The mean diameter of the specimens was 15 mm (20). Post-orchiectomy treatment choices for stage I seminoma are surveillance, adjuvant radiotherapy, or platinum-based chemotherapy (21). For the patient with bilateral seminoma or different histopathology surveillance is not recommended and radiotherapy or chemotherapy should be initiated. Follow-up includes physical examination, measurement of serum markers, chest radiographs, scrotal ultrasound and computed tomography of abdomen. Overall, patients with synchronous tumors present more advanced disease and have less favorable survival rates compared to those with metachronous tumors (6). In our case, the patient presented with a seminoma of stage IB on one testis and a mixed germ cell tumor of the same stage on the other testis. So, we decided to treat the patient with bilateral orchiectomy followed by one cycle of adjuvant BEP.

In conclusion concurrent bilateral testicular cancer of different histopathology is an exceedingly rare situation. Seminoma is the most common histopathologic type and there are only a few cases reported which the histopathology of the malignancies manifested in either of the testicles are different. The treatment is based on the clinical stage and the histopathological type of the tumor is determined by the most malignant component. Principles of treatment are similar, and it should not be considered that whether the histolopathology is the same or different in two testicles. Standard practice for these patients is bilateral radical orchiectomy followed by an adjuvant therapy upon the clinical stage and the pathological type. 
